# An Unexpected Presentation of Haemoperitoneum in a Pregnant Woman

**DOI:** 10.1155/2015/169582

**Published:** 2015-02-23

**Authors:** Kaushalya Arulpragasam, Andrea Atkinson, Mathias Epee-Bekima

**Affiliations:** King Edward Memorial Hospital, Bagot Road, Subiaco, WA 6008, Australia

## Abstract

In the majority of tertiary centres the Emergency Room or Assessment Unit is the gateway to the rest of the hospital. It is the location where critical decisions are formulated depending on whether a patient's condition is serious enough to warrant admission and, at times, emergency surgery. On occasion this decision can be straightforward based solely on the patient's presentation, observations, and basic investigations. This case highlights that although the decision and initial management may be apparent, often the diagnosis can be unexpected and that the diagnostic challenge is often outside the scope of a brief Emergency Room assessment. Corpus luteal cyst rupture is a common phenomenon but often not the cause of significant morbidity as it was in this case, especially in the absence of any associated risk factors.

## 1. Introduction

When assessing the “acute abdomen,” the top differential diagnosis for a female of reproductive age with acute onset abdominal pain is ectopic pregnancy. In addition, a positive quantitative b-HCG, last menstrual period eight weeks prior, and an ultrasound demonstrating an empty uterus and haemoperitoneum make an ectopic pregnancy increasingly likely. In this case although our management is unlikely to have been altered, the final diagnosis was unexpected.

## 2. Case Report

A twenty-six-year-old female, gravida two para one, was transferred to our tertiary gynaecological facility from the nearby general hospital with a presumptive diagnosis of a ruptured ectopic pregnancy. The patient presented in the early hours of the morning with sudden onset left iliac fossa pain, which woke her from sleep four hours prior. The patient was unsure of the date of her last menstrual period (LMP) but estimated that it was eight weeks prior and reported no per vaginal bleeding currently. In the time since initial presentation at the general hospital to her evaluation at our centre her haemoglobin had dropped 24 points from 132 g/L to 108 g/L and a bedside transabdominal ultrasound scan revealed approximately one litre of free fluid in the abdomen. An initial quantitative b-HCG was 14 IU/L and given the possibility of the patient's LMP being eight weeks prior a ruptured ectopic pregnancy was highly expected and the patient underwent an urgent laparoscopy. Apart from some recent illicit drug use in the form of methamphetamines and a normal vaginal delivery seven years previously the patient's medical history was unremarkable. She was not currently using any medical form of contraception.

**Figure 1:**
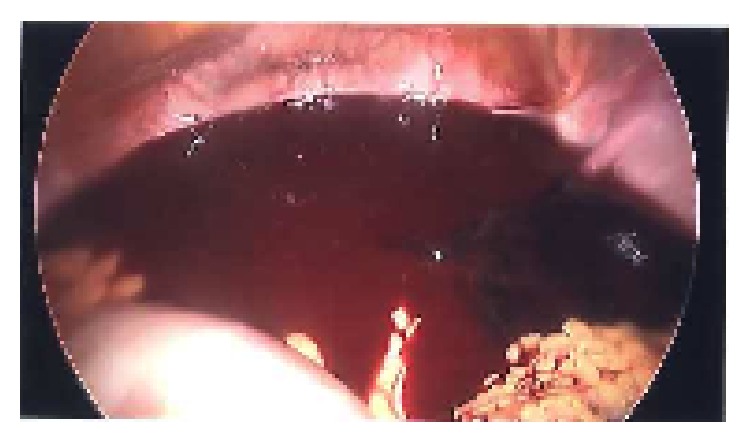
Pelvic haemoperitoneum and clots.

**Figure 2:**
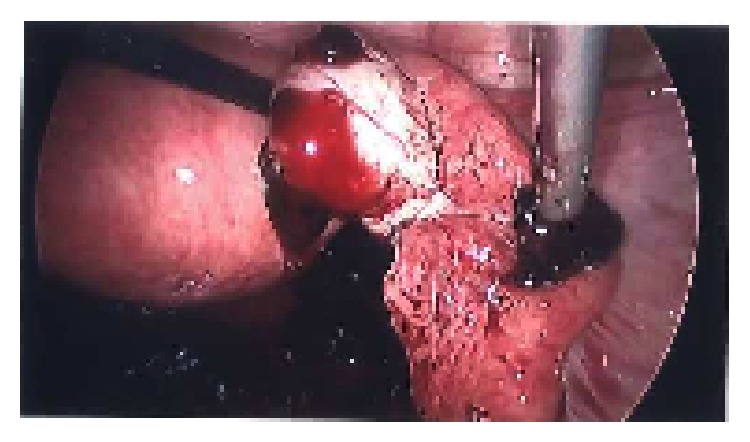
Only identifiable source of haemorrhage from corpus luteal cyst.

At laparoscopy one litre of blood was drained from the abdomen but no sign could be found of an ectopic pregnancy (see Figure 1). The uterus was of normal size, the fallopian tubes were unremarkable, and a small active bleeding source was identified on the right ovary but without an obvious cyst seen (see Figure 2). A limited amount of diathermy to this area controlled the bleeding. The upper abdomen inspection was normal. There was no hysteroscopy performed or handling of endometrium at laparoscopy. After removal of blood and organised clots from the abdomen laparoscopically the operation was concluded and the patient returned to the ward.

The next day the patient was discharged home as she was haemodynamically stable but two days later at a scheduled follow-up the quantitative b-HCG level was 38 IU/L, a clear doubling. The patient also reported mild pelvic pain and a small amount of per vaginal bleeding. In her next follow-up visit quantitative b-HCG level was 725 IU/L and the patient had ongoing per vaginal bleeding but no identifiable pregnancy on transvaginal ultrasound scan. She was advised that the possibility of a viable intrauterine pregnancy could not be excluded. On her final documented follow-up two days later the quantitative b-HCG level dropped to 218 IU/L and she had a small amount of ongoing vaginal bleeding. The final diagnosis in this case was of haemoperitoneum secondary to a presumed ruptured right corpus luteal cyst and failed pregnancy of unknown location, likely intrauterine (see Table 1).

## 3. Discussion

The corpus luteum is a functional cyst which develops in the luteal phase of the ovarian cycle and regresses into the corpus albicans when pregnancy does not occur [1]. Given its thin-walled vascular structure it is easily prone to haemorrhage [2]. Rarely the cyst-walled structure ruptures causing haemorrhage to spread into the peritoneal cavity resulting in a haemoperitoneum [3–5].

The most common gynaecological causes of spontaneous haemoperitoneum in a woman of child bearing age are ruptured corpus luteal cysts and an ectopic pregnancy [3]. Radiological studies have demonstrated that Ultrasonography has a high sensitivity in distinguishing an ectopic pregnancy from a corpus luteal cyst. It is suggested that the wall of an ectopic pregnancy is hyperechogenic and a corpus luteal cyst hypoechogenic relative to the endometrium. Moreover the presence of an anechoic internal echo pattern makes the diagnosis of a corpus luteal cyst more likely. Dopplers are less useful as they demonstrate a “ring of fire” appearance in both an ectopic pregnancy and corpus luteal cyst [4, 6].

Since Ultrasonography could be inconclusive in excluding an ectopic pregnancy, the evaluation of serum b-HCG levels would help differentiate an ectopic pregnancy from a corpus luteal cyst. However there are reports to suggest the coincidental presence of a corpus luteal cyst with an intrauterine pregnancy [7]. The latter is a clinical conundrum that can be most challenging for the clinician in determining treatment.

In unclear cases, some reports recommend a “wait and see” approach constituting serial b-HCG levels and repeat ultrasound imaging [7]. In our case the patient had a positive pregnancy test with a litre of blood in her abdomen. Hence surgical intervention was necessary as she was already on the path to becoming haemodynamically unstable.

Furthermore, the majority of cases reported in the literature have been in women with acquired coagulation abnormalities, haemoglobinopathies, or anticoagulants [4, 8, 9]. Our case is unique in that the patient had no associated risk factors. This case highlights the importance of considering haemoperitoneum secondary to corpus luteal cyst rupture, in a fit and healthy woman, as a differential diagnosis in the acute abdomen (see Table 2).

## 4. Conclusion

The distinction between normal early pregnancy and early pregnancy complications can be challenging. Haemoperitoneum from a ruptured corpus luteal cyst supporting pregnancy can be a life-threatening surgical condition. This case highlights the importance of considering a ruptured corpus luteal cyst in the differentials of an acute abdomen.

## Figures and Tables

**Table 1:** Summary of timeline and investigations.

Day	1	3	13	15
Symptoms	Acute abdomen	Small amount PV bleeding	Ongoing PV bleeding	Ongoing spotting

Ultrasound	Haemoperitoneum approx. 1 litre		Empty uterus, no adnexal masses, both ovaries seen	

Quant b-HCG IU/L	14	38	725	218

**Table 2:** Comparison of diagnostic criteria for ectopic versus corpus luteal cyst rupture.

Ruptured ectopic pregnancy	Corpus luteal cyst rupture
Positive b-HCG test (quantitative or qualitative)	+/− Positive urinary pregnancy test, low levels expected on quantitative b-HCG testing

Ultrasonographic Doppler finding of “ring of fire” appearance of pelvic cystic structure	Ultrasonographic Doppler finding of “ring of fire” appearance of pelvic cystic structure

Radiological evidence of pelvic mass or cyst with hyperechogenic wall	Radiological evidence of ovarian cyst with hypoechogenic wall +/− anechoic internal echo pattern
